# The effect of electroacupuncture combined with donepezil on cognitive function in Alzheimer’s disease patients: study protocol for a randomized controlled trial

**DOI:** 10.1186/s13063-017-2052-y

**Published:** 2017-07-03

**Authors:** Weina Peng, Jing Zhou, Min Xu, Qing Feng, Lulu Bin, Zhishun Liu

**Affiliations:** 1 Department of Acupuncture, Guang’anmen Hospital, China Academy of Chinese Medical Sciences, Beijing, 100053 China; 2 Department of Neurology, Xuan Wu Hospital, Capital Medical University, Beijing, 100053 China; 3 Beijing University of Chinese Medicine, Beijing, 100029 China

**Keywords:** Electroacupuncture, Donepezil, Alzheimer’s disease, RCT, Study protocol

## Abstract

**Background:**

Alzheimer’s disease is a progressive neurodegenerative disease. Although some of the current treatments offer some symptomatic relief, this disease cannot be cured at present. Electroacupuncture may be effective for Alzheimer’s disease for cognitive function, but the evidence for its effectiveness is still limited. The aim of this study is to evaluate the add-on effect of electroacupuncture to donepezil for improving the cognitive function of Alzheimer’s disease.

**Methods/design:**

A total of 334 participants with Alzheimer’s disease will be randomly assigned to either an electroacupuncture combined with donepezil group or a donepezil group with a ratio of 1:1. Participants in the electroacupuncture combined with donepezil group will receive electroacupuncture in addition to donepezil for 12 weeks and will keep taking donepezil for the following 24 weeks. Participants in the control group will take donepezil only. The primary outcome is the change from baseline in the total score of the Alzheimer’s Disease Assessment Scale-cognition at week 12. A follow-up will be conducted 24 weeks after the treatment.

**Discussion:**

We expect to verify the hypothesis that acupuncture in addition to donepezil is better than donepezil in improving the cognitive function of patients with Alzheimer’s disease. This trial has a limitation that participant blinding is impossible.

**Trial registration:**

Clinical Trials.gov: ID: NCT02305836. Registered on 13 November 2014.

**Electronic supplementary material:**

The online version of this article (doi:10.1186/s13063-017-2052-y) contains supplementary material, which is available to authorized users.

## Background

Alzheimer’s disease (AD) is a neurodegenerative disease characterized by an insidious onset and a progressive impairment of cognitive function [1]. The ongoing decline of cognitive function leads to a high risk of disability which is a significant burden for patients and caregivers around the world. The incidence of AD increases in people over 65 years of age, with a prevalence of 4.8% in this age group and representing approximately 60–80% of dementias [1, 2]. Moreover, the health costs associated with AD, the reduced quality of life, and the increased risk of mortality severely affects the social development and quality of family life of AD patients [3–5].

Although some of the current treatments offer some symptomatic relief, this disease cannot be cured at present [6]. Cholinesterase-inhibitors (CIs) donepezil, rivastigmine, and galantamine are recommended for clinical use according to the guideline of The National Institute for Clinical Excellence (NICE, now National Institute for Health and Clinical Excellence) [7]. The glutamate receptor antagonist memantine has also been approved and recommended by the Food and Drug Administration (FDA) to treat moderate to severe AD [8]. CIs have modest symptomatic but not curative effects [9] and these classes of drugs have considerable adverse reactions [10, 11]. Some studies [12–14] have shown that acupuncture may be effective for improving the cognitive function of AD patients; however, because of the lack of rigorous randomization and adequate sample size, there is still not enough evidence to show the effectiveness of acupuncture. This trial aims to determine the effectiveness of electroacupuncture (EA) as an adjuvant therapy for donepezil in improving the cognitive function of patients with AD.

## Methods/Design

**Fig. 1:**
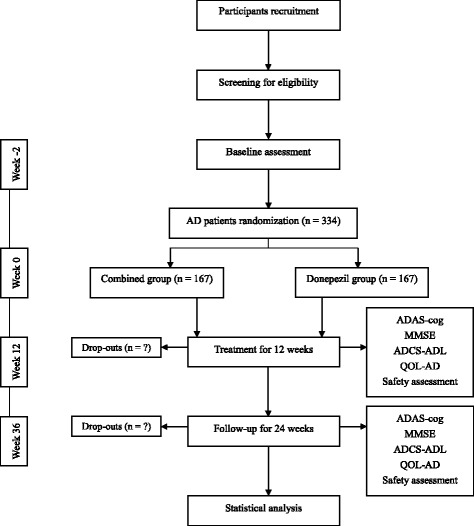
The flowchart of the trial

This trial is a prospective, randomized controlled, clinical trial to be conducted between June 2017 and December 2019. Three hundred and thirty-four participants with AD will be recruited at Guang’anmen Hospital, Beijing, China via the hospital web or posters. Recruiting and screening will be in the charge of a research assistant, whereas the diagnosis will be made by a neurologist. A 2-week baseline assessment will be conducted. Randomization is performed by the pharmacological assessment center at Guang’anmen Hospital. The participants identified as eligible will be randomized into two groups—EA combined with donepezil and donepezil—at a ratio of 1:1. The random block is set to 4, and random grouping is produced by using the Statistics Analysis System (SAS) software. Random numbers and information about grouping are sealed into opaque envelopes with correlative sequence numbers of entering the trial marked on the outside. Acupuncturists will open the envelopes according to the sequence numbers. The flowchart of the trial is shown in Fig. 1. The total study period of this trial is 38 weeks (Fig. 1), including 2-week baseline assessment, 12-week treatment period and 24-week follow-up period.

The trial will be conducted in accordance with the principles of the Declaration of Helsinki, and has been approved by the review boards and ethics committees of the participating hospital (Ethics approval number: 2014EC087).

## Participants

Participants meeting all of the following criteria will be included in this trial: (1) the diagnostic criteria of Neurological Communicative Disorders and Stroke and the Alzheimer Disease and Related Disorders Association (NINCDS-ADRDA) [15] or the Operational Criteria for the Diagnosis of Alzheimer’s Disease (OCDAD) [16], (2) aged between 60–85 years, (3) cognitive impairment based on the scores of the Chinese version of the Mini Mental State Examination (MMSE) (an illiteracy group ≤19, a primary school group ≤22, a junior high school and high school group ≤3, and a well-educated group ≤26) [17], (4) absence of depression (via an emotional assessment), (5) magnetic resonance imaging (MRI) confirmation of atrophy of the hippocampus or the medial temporal lobe volume, (6) the Medial Temporal Lobe Atrophy Rating Scale (MTA-scale) score (≥2 for those under 75 years, and ≥3 for those over 75 years).

Participants meeting any one of the following criteria will be excluded from this trial: (1) cognitive impairment caused by other factors (e.g., vascular dementia, dementia with Lewy bodies, frontotemporal dementia, hormone or metabolic abnormalities, hypothyroidism, folic acid or vitamin B_12_ deficiency, delirium or other mental and emotional disorders (such as schizophrenia and depression)), (2) a serious heart condition, hepatic disease, renal system disease, hematopoietic system disease, or whole-body malnutrition, (3) aphasia, disturbance of consciousness, or failure to cooperate with the related examinations due to physical disability, (4) anticoagulant treatments such as warfarin or heparin, (5) use of pacemakers, or (6) receiving acupuncture or CI treatment in the past 2 weeks.

### Intervention

#### EA combined with donepezil group

The acupoints are GV 16, GV 20, GV 24, and *Shang Yintang* (up to GV 29 1 cun), as well as bilateral EX-HN 5 and KI 4. The location of the acupoints is based on the *The Name and Location of Acupoints* drafted in 2006 by the National Standard of the People’s Republic of China (GB/T 12346-2006). The acupuncture protocol is based on reviews of acupuncture for AD [18, 19] published and expert consensus. Acupuncturists who have at least 2 years of practical experience will perform the intervention. Disposable, sterilized needles made of stainless steel (Hwato, Suzhou Medical Appliance Factory, Suzhou, China) and electric stimulators (SDZ-V electroacupuncture apparatus, Suzhou Medical Appliance Factory, Suzhou, China) will be used in the treatment group. After routine disinfection, the GV 16 will be needled while participants are in the sitting position with their head slightly forward and their muscles relaxed. The needle (25 mm in length) will be inserted obliquely and slowly downward toward the mandibular direction to a depth of 12.5 mm. The needle will be immediately removed after “*de qi*” (*de qi*, which is very important to therapeutic effect, is a sensation of soreness, heaviness, and numbness when needling [20]) and not retained. *Shang Yintang*, GV 20, and GV 24 will be needled (40 mm in length) at a 30° angle to a depth of approximately 12.5 mm while participants are in the decubitus position. For bilateral EX-HN 5 and KI 4, the needle (40 mm in length) will be vertically inserted to a depth of 25 mm. The electric stimulator will be applied to GV 20, GV 24, and bilateral EX-HN 5 with a spare-dense wave of 10/50 Hz, 0.5–5.0 mA. The current intensity is set up to the maximum tolerance of patients. There are three sessions per week with each session lasting for 30 min. There will be 12 weeks of treatment with 36 sessions for each participant in total. EA treatment will last for 12 weeks.

Donepezil (Eisai Pharmaceuticals, Shanghai, China) 5 mg will be given once daily before bed-time for the first 4–6 weeks. Based on the treatment effect, the dosage may be increased to 10 mg once daily before bed-time for the next 6–8 weeks. Donepezil will be taken for continuously for 24 weeks.

#### Donepezil group

Participants will only receive donepezil using the same administration method as with the EA combined with donepezil group and the therapy will last for 24 weeks.

Routine medications and therapies taken by participants with other complex chronic diseases will be recorded in detail in a paper Case Report Form. The intervention will be terminated due to severe adverse events.

### Outcome measures

The primary outcome is the change from baseline in the Alzheimer’s Disease Assessment Scale-cognition (ADAS-cog) [21] score measured at week 12. The maximum score of the ADAS-cog is 70 including 15 items of cognitive dysfunction assessment. The higher values indicate higher degree of deficit [22].

The secondary outcomes include the following items: the change from baseline in the ADAS-cog score measured at week 36. The change from baseline in total MMSE score [23], in the Alzheimer’s Disease Cooperative Study-Activity of Daily Living (ADCS-ADL) score, and in the Quality of Life-Alzheimer’s Disease (QOL-AD) score measured at weeks 12 and 36. The MMSE score is commonly used for estimating the severity of cognitive impairment with a maximum score of 30. The questions in this scale included orientation to time, orientation to place, attention and calculation, recall of three words, and visual construction. The ADCS-ADL score contains 19 domains about assessment of basic and operational ability of daily living [24]. The QOL-AD score, which includes physical health, mental health, social and financial assessment, and quality of life domains, is completed by both patients and caregivers [25].

All the adverse events will be recorded and assessed throughout the whole study period. Adverse reactions related to acupuncture may be fainting, hematoma formation, local infection or skin pain. Individuals who develop severe complications will be excluded from continuing in study, and the reasons for their termination will be recorded.

### Sample size and statistical analysis

The calculation of sample size is based on the primary outcome which is the change from baseline in the ADAS-cog score. According to previous studies [26], 39% of patients showed an improvement of at least 4 points on the ADAS-cog score after being treated with donepezil. We estimated the rate to be 39% in the donepezil group and 55% in the EA combined with donepezil group. Three hundred and four participants were needed to provide 80% power at a significant level of 5% using analysis of variance. The total sample size required for the study is 334 (167 each group) assuming a 10% loss to follow-up.

Baseline score of the ADAS-cog and age will be used as a covariate when assessing the differences between the EA combined with donepezil group and the donepezil group by an analysis of covariance for the primary and the secondary variables if data fits the normal distribution. Otherwise, a nonparametric test will be used to compare the statistical difference between the two groups. All the data will be blindly analyzed by the statisticians from the Clinical Evaluation Center of Guang’anmen Hospital. SPSS 20.0 (IBM Corp., Armonk, NY, USA) statistical software will be used for the statistical analyses. Subanalysis will be applied to the statistical analysis according to the severity of the condition of participants measured by the ADAS-cog [27]).

Efficiency and safety analysis will be based on all the participants at randomization according to the intention-to-treat (ITT) principle. Missing values assumed missing at random will be handled by the multiple imputation method [28]. Continuous data will be presented by the average and standard error. Two-sided tests will be used for all outcomes. A *P* value of less than 0.05 is considered to show statistical significance.

For the safety assessments, a description on the adverse events and adverse reactions will be presented in tables. These tables will include the categories, severity, rate of incidence, and correlation with the treatment. Individuals with serious adverse events should stop receiving the intervention and details will be reported in a timely manner.

### Quality control

All of the investigators participating in this trial will take a training course which lasts for 1 day. The assessment of all the scales will be in the charge of the research assistants who do not know the distribution of groups. Members of an independent Data Monitoring Committee (DMC) will regularly inspect the data and monitor the trial progression before the data statistics. Data management and statistical analysis will be under the control of the statisticians.

### Ethics and dissemination

**Fig. 2:**
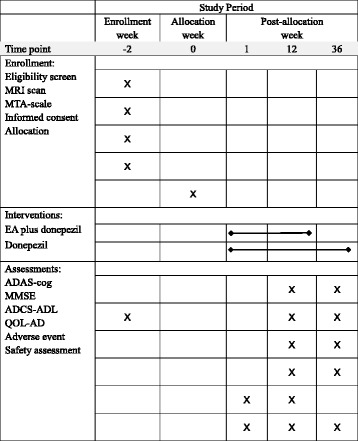
Standard Protocol Items: Recommendations for Interventional Trials (SPIRIT) figure. *MRI* Magnetic Resonance Imaging; *MTA-scale* Medial Temporal Lobe Atrophy Rating Scale; *EA* electroacupuncture; *ADAS-cog* Alzheimer’s disease Assessment Scale-cognition; *MMSE* Mini Mental State Examination; *ADCS-ADL* Alzheimer's disease cooperative study-activity of daily living; *Qol-AD* Quality of life-Alzheimer’s disease

This study has been approved by the Ethics Committee of Guang’anmen Hospital, CACMS (2015EC043). This protocol was written following the Standard Protocol Items: Recommendations for Interventional Trials (SPIRIT) Checklist (Additional file 1) and figure (Fig. 2). All important protocol modifications will be submitted to the Ethics Committee of Guang’anmen Hospital. All participants and caregivers will be informed about the potential risks and benefits of the study. Informed consent will be obtained for either all participants or, if their capacity to consent is absent, their nearest relatives or their caregivers. All personal information about participants will be prohibited from other use and stored in specific cabinets in order to protect confidentiality before, during, and after the trial. Statistical and monitoring managers will have access to the final trial dataset. For compensation to those who suffer harm from trial participation, we will provide free corresponding therapies and medical consultation. The results of this trial are planned to be disseminated in conferences or publications.

## Discussion

In recent years, previous studies have shown that acupuncture might be effective in improving the cognitive function of AD patients [29, 30]. The results of this study will contribute to a better understanding that whether EA has additional effects to donepezil for improving the cognitive function of patients with AD.

Although clinical diagnosis is the core criteria for AD patients, preclinical diagnostic criteria, such as bio-marker evidence for AD, are also used in clinical practice and research [17]. Medial temporal lobe atrophy (MTA) as the typical imaging appearance can differentiate AD from ageing with a sensitivity and specificity of 80–85%. The severity of MTA combined with clinical information seems justified to be taken into account when diagnosing AD in clinical practice [31]. Therefore, the included criteria of this trial are based on the degree of atrophy of hippocampal or medial temporal lobe volume (measured by MRI). Moreover, as the MTA-scale scores differentiate between moderate to severe AD patients with a sensitivity of 70–100% and a specificity of 67–96%, according to the severity of the condition of participants, subgroup analysis will be applied in the statistical analysis. Furthermore, donepezil is approved by the FDA to treat the symptoms of mild to moderate AD [24, 25] and studies have shown that it can improve the neuropsychiatric, cognitive, and global functions [32–34]. Accordingly, donepezil was chosen as a positive control in this trial. Additionally, an appraisal of drug evolution for AD has shown that the primary outcomes of trials can be described within cognitive, functional, global change, and severity domains [17]. On the basis of this appraisal, the primary outcome is the ADAS-cog score. The ADAS-cog, assessing the severity of impairment of selective areas of cognition, is a sensitive and reliable neuropsychological test [35]. Moreover, a previous trial [36] which had a similar design also evaluated the change in ADAS-cog score after treatment, but follow-up effectiveness was not assessed. Therefore, a 12-week treatment with a follow-up at 24 weeks is expected to better demonstrate the additional effect of EA to donepezil in treating AD.

However, a limitation of this trial is that participant blinding is not possible due to the two different therapeutic strategies employed for each group. Therefore, to minimize any bias that may result from the two treatments, the process of randomization will be under strict control and the investigators and data analysts are to be blinded. Acupuncture as a complex intervention produced greater response expectancies and placebo effects [37, 38]. However, the expectancy effects of acupuncture cannot be estimated in this trial due to the impaired cognitive function of AD patients.

### Trial status

We are currently recruiting participants for this trial.

## Additional file

Additional file 1: SPIRIT Checklist. (DOCX 21 kb)

## Abbreviations

AD: Alzheimer’s disease
ADAS-cog: Alzheimer’s Disease Assessment Scale-cognition
ADCS-ADL: Alzheimer’s Disease Cooperative Study-Activities of Daily Living
CIs: Cholinesterase inhibitors
DMC: Data Monitoring Committee
EA: Electroacupuncture
FDA: Food and Drug Administration
ITT: Intention-to-treat
MMSE: Mini Mental State Examination
MRI: Magnetic resonance imaging
MTA-scale: Medial Temporal Lobe Atrophy Rating Scale
NICE: National Institute for Health and Clinical Excellence
NINCDS-ADRDA: Neurological Communicative Disorders and Stroke and the Alzheimer Disease and Related Disorders Association
OCDAD: Operational Criteria for the Diagnosis of Alzheimer’s disease
QOL-AD: Quality of Life-Alzheimer’s Disease
SAS: Statistics Analysis System
